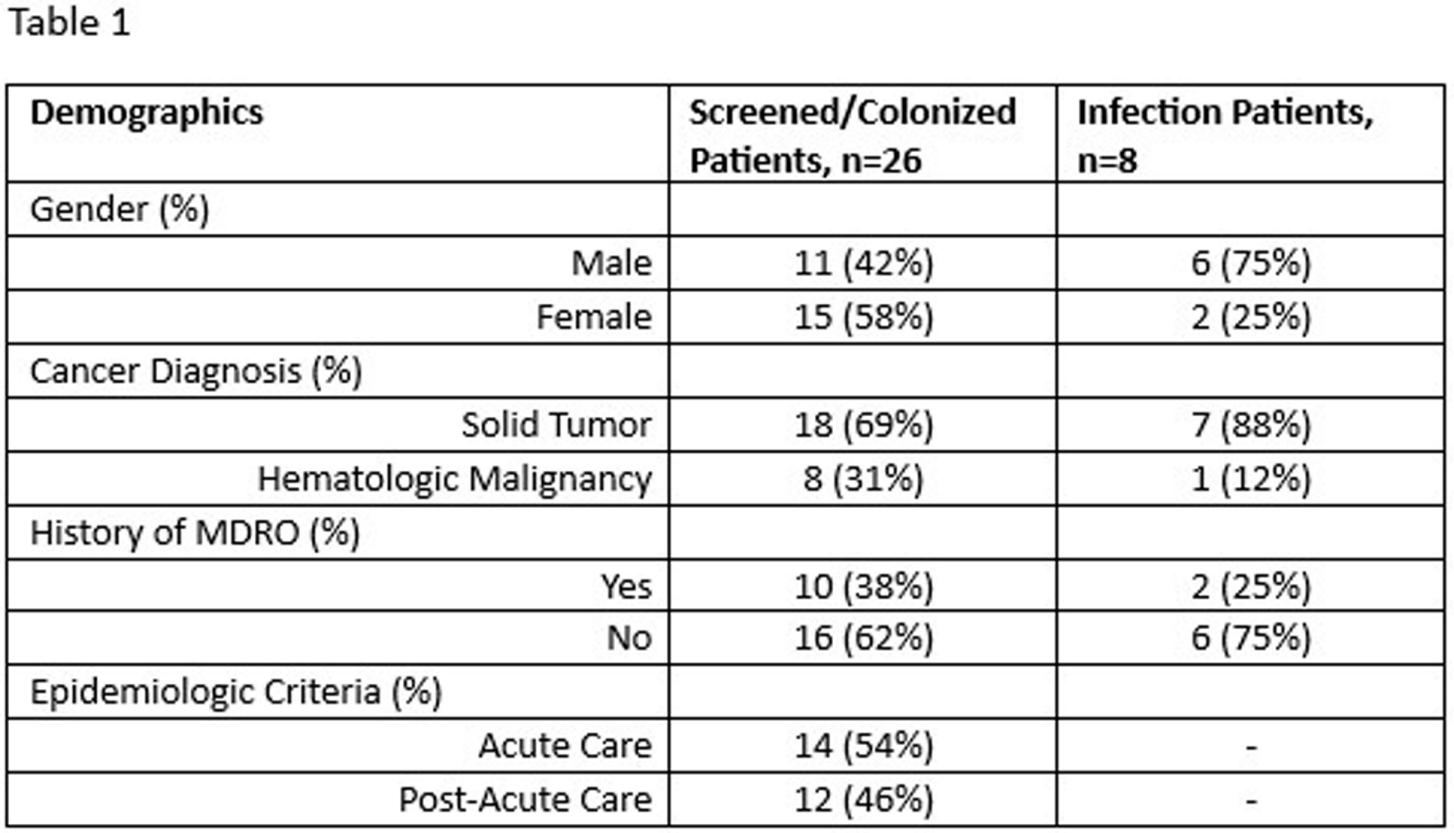# 228 Synovial Fluid PCR Testing Paired with Culture Can Improve Pathogen Identification

**DOI:** 10.1017/ash.2026.10607

**Published:** 2026-06-23

**Authors:** Adina Feldman, Micah Bhatti, Jane Powell, Guy Handley, Amy Spallone

**Affiliations:** 1 MD Anderson Cancer Center

## Abstract

**Background:** Candida auris (C. auris) is an emerging multidrug-resistant fungal pathogen that continues to spread across the United States and remains classified by the Centers for Disease Control and Prevention as an urgent public health threat. Due to its ability to persist on surfaces and spread easily in healthcare settings, early detection and rapid infection control measures are critical to prevent outbreaks. Based on surveillance data, we implemented targeted C. auris screening in 2024 for patients transferred from outside healthcare facilities. After one year, we assessed adherence, positivity, and clinical infection cases to determine our program’s success and identify potential gaps in screening criteria. **Methods:** All eligible transfer patients received composite axilla/groin swabs on admission that were sent for external laboratory Polymerase Chain Reaction (PCR) testing. Demographic, clinical, and epidemiological factors, including transferring healthcare facility origin and co-colonizing multidrug-resistant organisms (MDROs), were evaluated alongside clinical C. auris infections identified outside of admission screening (e.g., urine, blood, pleural fluid). These were used to compare characteristics between colonized and infected patients and determine whether expanded screening criteria were warranted. All C. auris-positive patients were placed on transmission-based precautions with rapid notifications to unit leadership. **Result:** Over the first year (Sept 2024–Sept 2025), 1,725 of 1,937 eligible patients were screened (89.1%), with a positivity rate of 1.50% (26 colonized patients). Patient refusal and screening cancellations were infrequent (18 events; 0.06%). An additional 212 eligible patients (11%) were not screened due to operational factors, with no subsequent positive C. auris clinical cultures during hospitalization. Eight additional patients were diagnosed with C. auris infection through clinical cultures. These patients were ineligible for screening as they were not transfers, though most had frequent readmissions to and from outside healthcare facilities. Demographic and clinical characteristics of colonized and infected patients are summarized in Table 1. **Conclusion:** Our findings demonstrate that a targeted admission screening program for interfacility transfers identified colonized C. auris patients and enabled timely implementation of infection control measures. However, patients with significant outside healthcare exposure who are not interfacility transfers remain high-risk, indicating a gap in our current screening criteria.

**Figure:**